# *Glial Cell*-*Derived Neurotrophic Factor* Functions as a Potential Candidate Gene in Obstructive Sleep Apnea Based on a Combination of Bioinformatics and Targeted Capture Sequencing Analyses

**DOI:** 10.1155/2021/6656943

**Published:** 2021-02-18

**Authors:** Yuanyuan Cao, Qing Zhu, Xintian Cai, Ting Wu, Xiayire Aierken, Ayguzal Ahmat, Shasha Liu, Nanfang Li

**Affiliations:** ^1^Xinjiang Medical University, Urumqi, 830001 Xinjiang, China; ^2^Hypertension Center of People's Hospital of Xinjiang Uygur Autonomous Region, Xinjiang Hypertension Institute, National Health Committee Key Laboratory of Hypertension Clinical Research, Urumqi, 830001 Xinjiang, China

## Abstract

**Background:**

Obstructive sleep apnea (OSA) is a prevalent chronic disease that increases the risk of cardiovascular disease and metabolic and neuropsychiatric disorders, resulting in a considerable socioeconomic burden. The present study was aimed at identifying potential key genes influencing the mechanisms and consequences of OSA.

**Methods:**

Gene expression profiles associated with OSA were obtained from the Gene Expression Omnibus (GEO) database. Differentially expressed genes (DEGs) in subcutaneous adipose tissues from patients with OSA and normal tissues were screened using R software, followed by gene ontology and pathway enrichment analyses. Subsequently, a protein-protein interaction (PPI) network was constructed and hub genes were extracted using Cytoscape plugins. The intersected core genes derived from different topological algorithms were considered hub genes, and the potential candidate gene was selected from them for further analyses of expression variations using another GEO dataset and targeted capture sequencing in 100 subjects (50 with severe OSA and 50 without OSA).

**Results:**

A total of 373 DEGs were identified in OSA samples relative to normal controls, which were primarily associated with olfactory transduction and neuroactive ligand-receptor interaction. Upon analyses of nine topological algorithms and available literature, we finally focused on glial cell-derived neurotrophic factor (GDNF) as the candidate gene and validated its low expression in OSA samples. Two rare nonsynonymous variants (p.D56N and p.R93Q) were identified among the 100 cases through targeted sequencing of GDNF, which could be potentially deleterious based on pathogenicity prediction programs; however, no significant association was detected in single nucleotide polymorphisms.

**Conclusion:**

The present study identified GDNF as a promising candidate gene for OSA and its two rare and potentially deleterious mutations through a combination of bioinformatics and targeted capture sequencing analyses.

## 1. Introduction

Obstructive sleep apnea (OSA) is a sleep-related respiratory disease characterized by breathing problems at night such as snoring, apnea, and daytime sleepiness, which leads to intermittent hypoxemia, transitory hypercapnia, and sleep structure disturbances. The principal clinical risk associated with OSA is multiple organ system impairment, such as cardiocerebrovascular diseases, metabolic syndrome, and cognitive dysfunction [1–3]. The prevalence of OSA has increased among the general population, and moderate to severe OSA has been estimated to be as high as 49.7% in males and 23.4% in females [4]. Unfortunately, the vast majority of patients with OSA (70*–*90%) remain undiagnosed, resulting in a heavy health and socioeconomic burden [5]. Therefore, it is imperative to study the etiology and pathogenesis of OSA and to identify indicators for early diagnosis and treatment targets.

Strong genetic influence has been reported for OSA, with more than 1.5-fold increased risk in first-degree relatives of patients [6]. Approximately 35% to 40% of variations in apnea-hypopnea index (AHI), which determines apnea severity, can be explained by genetic factors [7]. Genetic research has provided new insights into understanding the underlying mechanisms of OSA to develop a more effective targeted therapy and optimize treatment strategies. To date, most efforts focusing on identification of the genetic causes of OSA have used the candidate gene approach, which is based on four intermediate pathogenic pathways: obesity and body fat distribution, craniofacial morphology, ventilatory control, control of sleep, and circadian rhythm [7, 8]. However, the method limits validity of the findings because such studies depend on prior hypotheses on disease mechanisms, which precludes the determination of genetic variations in previously unknown pathways [9]. Only a few genome-wide association studies (GWAS) on OSA, which could provide further information with regard to the candidate gene approach, have been conducted to date [9–11]. However, data obtained from such study designs are currently limited. Furthermore, the findings on significant or potentially relevant genes that could influence susceptibility to OSA have been controversial, and the results from candidate gene studies and GWAS have not been consistent [8–11].

Recently, a genome-wide DNA microarray based on high-throughput platforms for gene expression analysis emerged as an efficient and relatively economical tool for studying the genetic basis of complex diseases [12]. In addition, the recent application of next-generation sequencing technologies, such as targeted capture sequencing, could be very instrumental in the identification of new pathogenic genes or new pathogenic sites of known pathogenic genes [13]. In the present study, we made the first attempt to identify susceptibility gene/locus for OSA by combining bioinformatics and targeted capture sequencing. We compared gene expression profiles in subcutaneous adipose tissues of patients with OSA and control samples obtained from the Gene Expression Omnibus (GEO) database to screen for differentially expressed genes (DEGs). Subsequently, the DEGs were identified using a combination of functional enrichment and protein-protein interaction (PPI) network analyses. Hub genes were identified based on the different topological algorithms and a potential candidate gene was selected from the hub genes for validation of gene expression using an additional independent dataset. Moreover, the associations between common and rare functional variants in the candidate gene with OSA were explored using targeted sequencing analysis to reveal potential genetic variants of OSA.

## 2. Materials and Methods

### 2.1. Microarray Dataset Source

A systematic retrieval of gene expression microarray datasets from the National Center for Biotechnology Information GEO (http://www.ncbi.nlm.nih.gov/geo/) was performed to evaluate DEGs between OSA and normal samples. The keyword “obstructive sleep apnea” was used for the screening. The gene expression profile of GSE135917 (https://www.ncbi.nlm.nih.gov/geo/query/acc.cgi?acc=GSE135917) [14] was included in the present study and was based on GPL6244 platform (HuGene-1_0-st; Affymetrix Human Gene 1.0 ST Array). The dataset contained 18 subcutaneous adipose tissue samples obtained from abdominal subcutaneous fat biopsies of 10 patients with OSA and 8 normal controls during a ventral hernia repair surgery [14]. OSA severity was assessed using the ARES™ Unicorder (Watermark Medical®, Boca Raton, FL, USA), a previously validated portable sleep monitor worn for two consecutive nights prior to surgery [14].

An additional independent dataset, GSE75097 (https://www.ncbi.nlm.nih.gov/geo/ query/acc.cgi?acc=GSE75097), which included microarray data of peripheral blood mononuclear cells in patients with OSA and primary snoring, was used to validate the results obtained from the GSE135917 dataset. The GSE75097 dataset on the GPL10904 platform comprised samples from subjects with primary snoring (PS; AHI < 5, *n* = 6), treatment-naïve patients with moderate to severe OSA (MSO; 15 < AHI ≤ 50, *n* = 16), and treatment-naïve patients with very severe OSA (VSO; AHI > 50, *n* = 12) [15].

### 2.2. Data Preprocessing and Differential Expression Analysis

We used the R software (version 3.6.2; https://www.r-project.org/) in addition to packages available from Bioconductor (http://www.bioconductor.org/) to perform statistical analyses. Processing, normalization, and quality control of the microarray datasets with raw data (.CEL files) were performed using the Affy package [16] in R on the Affymetrix platform. The robust multichip average (RMA) method was used for background correction, quantile normalization, and median polish summarization. Probe IDs were converted into gene symbols based on the annotation platform. Probe sets without corresponding gene symbols or genes with more than one probe set were removed or averaged.

The Linear Models for Microarray Data (LIMMA) package [17] in R Bioconductor was used to identify DEGs (∣log_2_ fold change | ≥1; false discovery rate (FDR) < 0.05) between OSA samples and normal controls. FDR was controlled using the Benjamini-Hochberg method, and empirical Bayes-modified *t*-tests were performed to select sets of DEGs. Heat map and volcano plot of DEGs were generated in R using the ggplot2 and pheatmap packages.

### 2.3. Biological Functions and Pathway Enrichment Analyses

The identified DEGs were subjected to gene ontology (GO) term enrichment and Kyoto Encyclopedia of Genes and Genomes (KEGG) pathway analyses using clusterProfiler package (version 3.10.0) [18] and org.Hs.eg.db annotation package (version 3.10.0) [19] in R to further elucidate the biological functions of DEGs identified in OSA and normal control samples. The significant GO terms or KEGG pathways were enriched by more than two genes with a threshold of FDR < 0.05.

### 2.4. Protein-Protein Interaction Network Analysis and Hub Gene Selection

Potential interactions among the DEGs encoding proteins were predicted with the aid of the online database, Search Tool for the Retrieval of Interacting Genes/Proteins (STRING version 11.0; http://string-db.org), and a combined interaction score > 0.4 was considered statistically significant. Subsequently, the PPI network was visualized using Cytoscape (version 3.7.2; http://www.cytoscape.org/). Molecular Complex Detection (MCODE; version 1.6), which is a plugin of Cytoscape, was used to search for the most dense and significant module in the PPI network using the following criteria: degree cutoff = 2, node score cutoff = 0.2, *K* − core = 2, and max depth = 100. cytoHubba (version 0.1), a Cytoscape plugin was used to select and identify the top ten ranking genes based on 11 topological analysis algorithms [20]. The top ten nodes were defined as core genes for each algorithm in the topological network. The intersected core genes derived from nine topological algorithms were considered as hub genes. The intersection of the top gene sets revealed the hub genes, which were presented using a Venn diagram generated with OmicShare tools (http://www.omicshare.com/tools). Furthermore, functional annotation of hub genes was carried out using Database for Annotation, Visualization and Integrated Discovery (DAVID database version 6.8; https://david.ncifcrf.gov/). The potential candidate was selected from the hub genes based on available literature and through data mining.

### 2.5. Validation and Analysis of the Candidate Gene

An additional independent microarray dataset, GSE75097, was used to verify the differential expression of the candidate genes in OSA and control samples. The analyses and visualization of gene expressions were performed using ggpubr and ggplot2 R packages. The DEGs (*P* value < 0.05) were used for subsequent targeted sequencing.

### 2.6. Targeted Capture Sequencing of the Candidate Gene

#### 2.6.1. Ethics Statement

The present study complied with the Declaration of Helsinki and was approved by the Local Ethics Committee of the People's Hospital of Xinjiang (Xinjiang, China). Written informed consent was obtained from all subjects prior to participation in the study.

#### 2.6.2. Subjects

Subjects referred to the Hypertension Center of the People's Hospital of Xinjiang for polysomnography (PSG) as an initial diagnosis of OSA were recruited consecutively for the study from April to December 2016 (previously described in [21]). Subjects without OSA and severe OSA (exhibiting extreme phenotype based on AHI) were enrolled for an additional genomic study. In the present study, we selected AHI (either very low or very high) as the extreme phenotype. Extreme phenotype sampling that involved selection of subjects from the extremes of trait distribution was applied, which could enhance the ability and statistical power to detect rare variants. Following the inclusion and exclusion criteria as detailed in our previous study [21], while excluding smokers, a total of 100 subjects (50 without OSA and 50 with severe OSA) were enrolled for the study.

#### 2.6.3. Interventions

Overnight PSG monitoring, clinical data acquisition, blood sample collection, and genomic DNA extraction from all subjects were performed according to the standard procedures as previously described [21, 22]. All subjects underwent overnight PSG monitoring (Compumedics E series, Australia) and were evaluated by a registered polysomnographic technologist based on the American Academy of Sleep Medicine criteria for scoring sleep and associated events [23]. Subjects without OSA were defined as those having an AHI of <5 events/h, and subjects with severe OSA had an AHI of ≥30 events/h. The general demographic and clinical data, which primarily included age, gender, body mass index (BMI), neck circumference, and abdominal circumference, as well as personal/family medical history and lifestyle habits (alcohol consumption, smoking status), were collected. Two equal samples of fasting venous blood (fasting ≥ 10 h, cubital vein blood sample) were collected from each subject in the morning after PSG. Serum biochemical parameters were determined using an automatic biochemical analyzer (Beckman Coulter, CA, USA) at the central laboratory of the People's Hospital of Xinjiang according to standard procedures. Venous blood samples (3 ml) from all subjects were collected in EDTA anticoagulant tubes. Genomic DNA was extracted from whole blood using PAXgene Blood DNA Kit (Qiagen, Hilden, Germany), and the purity of DNA was measured using a spectrophotometer (NanoDrop 2000; Thermo Fisher Scientific Inc., Waltham, MA, USA). The total amount of DNA required from all samples was at least 2 *μ*g with a concentration of DNA ≥ 50 ng/*μ*L. Afterwards, the extracted DNA was preserved at -80°C and sent to Genesky Biotechnologies Inc. (Shanghai, China) for targeted capture sequencing.

#### 2.6.4. Targeted Capture Sequencing

DNA samples (*n* = 100) were analyzed through targeted capture sequencing of the key hub genes using Agilent SureSelect^XT^ custom Kit (Agilent Technologies, Santa Clara, CA, USA) on an Illumina HiSeq platform (Illumina, San Diego, CA, USA). Sequencing reads were aligned to the human reference genome (UCSC hg38) using BWA algorithm, and variant calling was carried out using GATK HaplotypeCaller. Single-nucleotide variant annotation was performed with Annotate Variation (ANNOVAR; http://annovar.openbioinformatics.org/en/latest/). Frequency of variants was evaluated based on publicly available databases (1000 Genomes, ESP6500, and ExAC03). A combination of pathogenicity prediction software packages (SIFT [24], POLYPhen V2 [25], MutationTaster [26], CADD [27], and DANN [28]) was used to predict the potential impact of each genetic variant on gene function. Requiring at least two of the software packages to support the variant may be damaging.

## 3. Statistical Analyses

Continuous data were expressed as means ± standard deviations or medians (interquartile range), while categorical data were expressed as *n* (%). Independent Student's *t*-test or Mann-Whitney *U*-test was used to analyze continuous variables based on the normality of data distribution. Chi-squared test or Fisher's exact test was used to analyze categorical variables as appropriate. Hardy–Weinberg equilibrium, single nucleotide polymorphism (SNP) association analyses, and multiple comparison correction were performed using PLINK (version 1.0.7; http://pngu.mgh.harvard.edu/ purcell/plink/). A *P* value < 0.05 was considered statistically significant.

## 4. Results

### 4.1. Identification of DEGs

**Figure 1:**
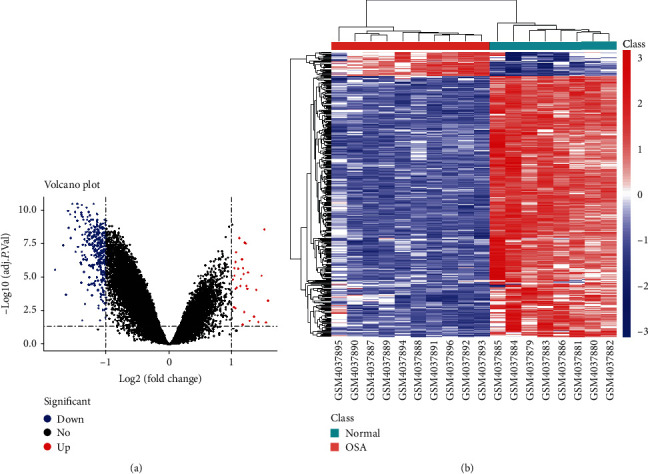
DEG screening and visualization for GSE135917. (a) Volcano plot of the DEGs for OSA samples vs. normal controls. Each dot represents a gene (red: upregulated gene; blue: downregulated gene; black: no significant difference). (b) Heat map and clustering pattern of the DEGs including 342 downregulated and 31 upregulated genes. Red or blue indicates either higher or lower expression levels of DEGs. Samples were separated into the normal and OSA cluster.

Raw microarray data from the GSE135917 dataset was subjected to RMA preprocessing and subsequently normalized to the median of all samples. A box plot of each sample before and after normalization demonstrated that the chip data had been normalized and were available for DEG selection (Supplementary Material 1, Supplemental Figure S1). A total of 23281 gene expression values were obtained from the 18 samples after data preprocessing. Based on the calculation criteria of absolute log_2_FC ≥ 1 and FDR < 0.05, 373 DEGs, which included 342 downregulated genes and 31 upregulated genes were identified in OSA samples relative to normal controls (Supplementary Material 2). A volcano plot (Figure 1(a)) was used to illustrate the distribution of DEGs, and a heat map (Figure 1(b)) used to present the results of bidirectional hierarchical clustering of DEGs and samples based on the expression level of DEGs.

### 4.2. Functional and Pathway Enrichment Analyses of DEGs

**Figure 2:**
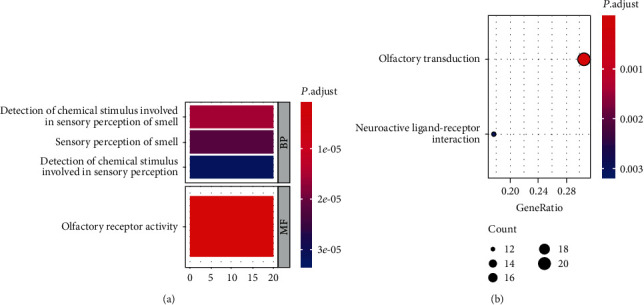
Enrichment analysis of the DEGs using clusterProfiler R package: (a) GO enrichment analysis; (b) KEGG pathway analysis. Four significant GO terms (3 BP and 1 MF) and two KEGG pathways were identified with the cutoff criteria of adjusted *P* value < 0.05. GO: gene ontology; KEGG: Kyoto Encyclopedia of Genes and Genomes; BP: biological process; MF: molecular function.

GO term enrichment and KEGG pathway analyses were performed using the clusterProfiler package in R, and only the GO terms and KEGG pathways enriched with an adjusted *P* value < 0.05 (Benjamini-Hochberg correction for multiple testing) were considered. The GO functional enrichment analysis results revealed that 373 DEGs were mapped to four GO terms including three biological process (BP) terms and one molecular functional (MF) term (Figure 2(a)). Enriched GO terms were predominantly associated with olfactory receptor activity and detection of chemical stimulus involved in sensory perception of smell. Furthermore, KEGG pathway analyses demonstrated that DEGs were significantly enriched in olfactory transduction and neuroactive ligand-receptor interaction pathways (Figure 2(b)).

### 4.3. PPI Network Analysis and Hub Gene Selection

**Figure 3:**
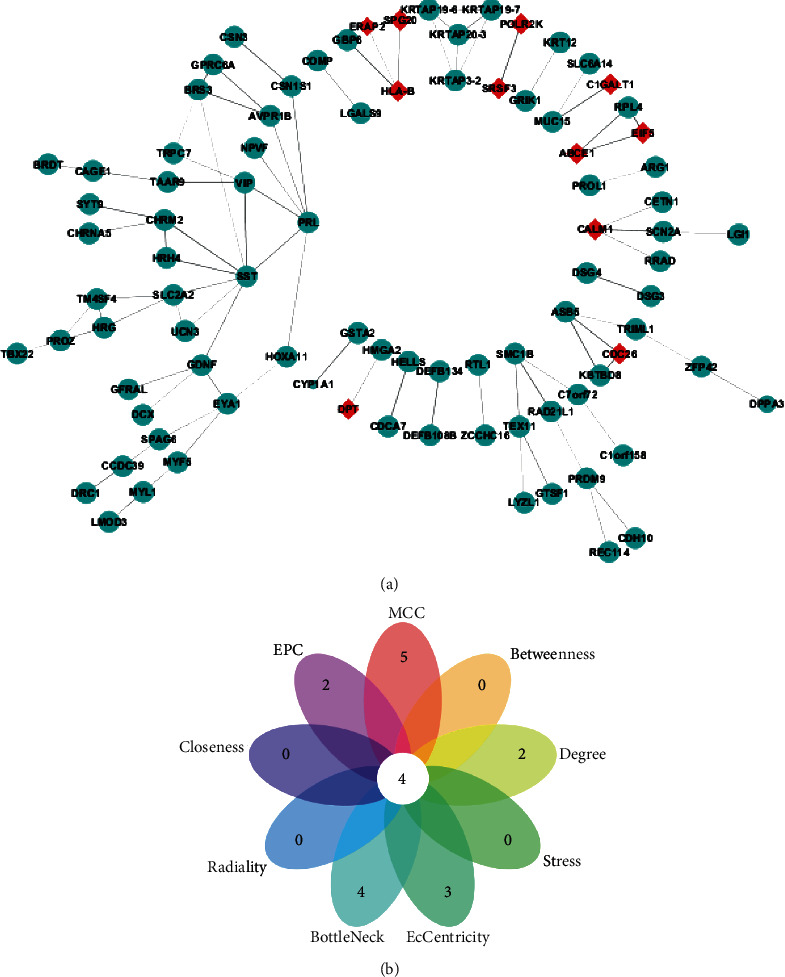
PPI network analysis and hub gene selection. (a) PPI network of the DEGs. Blue circles and red diamonds represent downregulated genes and upregulated genes, respectively; the size of circles or diamonds indicates *P* values, with larger circles or diamonds representing smaller *P* values. (b) Venn diagram of gene sets ranking top ten based on nine topological analysis algorithms in cytoHubba plugin. The intersection of the top gene sets revealed four hub genes. PPI: protein-protein interaction.

A PPI network comprising 90 nodes and 86 edges was constructed based on the biological interactions of 373 DEGs to further identify their associations at the protein level (Figure 3(a)). We screened 11 upregulated and 79 downregulated genes in patients with OSA. Subsequently, we combined the results of MCODE and cytoHubba analyses and selected the top ten ranking genes based on nine topological analysis algorithms (Table 1). A Venn diagram-based analysis was used to determine the intersection of the nine gene sets, as presented in Figure 3(b). Finally, the key hub genes including glial cell line-derived neurotrophic factor (GDNF), SLC2A2, PRL, and SST were identified and are presented in Supplementary Material 1 (Supplemental Table S1); a functional annotation of the genes was processed using DAVID. Strikingly, the hub gene, GDNF, has been previously reported to be a key candidate gene associated with OSA in a large candidate gene study [8], but it remains controversial. Combining our data mining analyses, we finally focused on the GDNF gene and used a different independent dataset to validate GDNF gene expression.

### 4.4. Validation of GDNF Gene Expression

**Figure 4:**
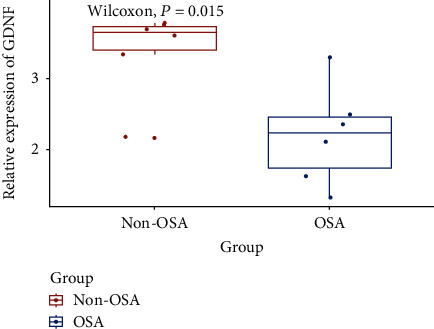
Validation of GDNF gene. Differential expression of GDNF gene in non-OSA and OSA samples based on GSE75097. GDNF: glial cell-derived neurotrophic factor.

Gene expression level analysis of GDNF based on data obtained from an independent dataset, GSE75097, was performed to validate the reliability of the results obtained from the GSE135917 dataset. In the validation dataset, the non-OSA group, which comprised of six subjects with primary snoring, and an equal number of patients with OSA selected from the MSO or VSO groups based on age, gender, and major comorbidities were included in the analyses (see Supplementary Material 1, Supplemental Table S2 for details). A comparison of baseline data of enrolled subjects from the GSE75097 dataset is summarized in Table 2. The expression level of the GDNF gene was significantly lower in the OSA group than in the non-OSA group (*P* = 0.015), which was consistent with the results obtained from the GSE135917 dataset (Figure 4). Therefore, we speculated that the GDNF gene could be an indicator of OSA and further applied the targeted capture sequencing technology to explore the association between common and rare variants in the GDNF gene with OSA.

### 4.5. Targeted Capture Sequencing of GDNF Gene

The clinical characteristics of subjects without OSA and patients with severe OSA are summarized in Table 3. The severe OSA group had more male, older, and obese individuals than the non-OSA group. Patients with OSA had a significantly higher BMI, abdominal and neck circumference, apnea index, hypopnea index, and AHI, as well as lower oxygen saturation than the patients without OSA (all *P* < 0.001). In addition, no significant differences were observed in alcohol consumption (*P* = 0.086), systolic blood pressure (SBP; *P* = 0.748), diastolic blood pressure (DBP; *P* = 0.389), sleep efficiency (*P* = 0.329), or total sleep time (*P* = 0.608) between the two groups.

The target region of GDNF gene was sequenced at an average depth of 411X in the present study. Based on the minor allele frequency (MAF) of the control group, all variations were designated as common (MAF > 1%) or rare (MAF < 1%). A total of 28 SNPs and one short tandem repeat were present in the GDNF gene fragment and were subjected to association analyses under additive, dominant, recessive, and allele genetic models using unconditional logistic regression models. All SNPs satisfied the Hardy–Weinberg equilibrium. The genotype and allele distributions of the 29 polymorphisms between the two groups under the four genetic models were not significantly different. However, we identified two rare nonsynonymous variants (p.D56N and p.R93Q), which could be potentially deleterious based on the prediction by SIFT, POLYPhen V2, MutationTaster, and DANN software packages (Table 4).

## 5. Discussion

Currently, bioinformatics has become increasingly crucial in the study of the pathogenesis of multifactorial disorders [29]. The identification of variations in gene expression in tissues relevant to a disease is a key step toward enhancing our understanding of pathogenesis and could eventually lead to improved diagnosis and treatment [29]. In the present study, we identified 31 upregulated and 342 downregulated genes in subcutaneous adipose tissues of patients with OSA when compared with normal controls, which suggested that the occurrence and development of OSA are a complex biological process involving multiple genes and steps. Biological function and pathway enrichment analyses indicated that DEGs were primarily involved in the GO terms or KEGG pathways associated with olfactory receptor activity and olfactory transduction, as well as neuroactive ligand-receptor interaction.

Furthermore, GDNF, SLC2A2, PRL, and SST were identified as the hub genes from the PPI network. Of note, GDNF, which has been previously reported to be a key candidate gene associated with OSA [8], was also screened and initially validated in our microarray data mining. Nevertheless, the association between GDNF and OSA remains controversial [8, 30] and warrants further studies. As such, the association between genetic variants of the GDNF gene with OSA was further explored using targeted capture sequencing. Although we could not find significant SNPs, we observed two rare nonsynonymous variants. The c.166G>A mutation results in an Asp-to-Asn amino acid change (p.D56N) of a conserved Asp, and the c.278G>A mutation results in an Arg-to-Gln amino acid change (p.R93Q) of a conserved Arg residue in GDNF, which has not been previously implicated in OSA. The mutation could influence the biochemical properties of proteins, which requires further functional studies and validation.

GDNF is a member of the transforming growth factor family, which is crucial for motor neurons, dopaminergic neurons, and peripheral neurons [31]. A large candidate gene study on OSA suggested a potential pathogenic role of polymorphisms in the GDNF gene (rs2910705, rs2975100, rs2973042, and rs2973041) in European Americans [8]. The associations of GDNF with OSA remained after adjustment for BMI, implying that the genetic variants influence susceptibility to OSA through obesity-independent pathways [8]. Larkin et al. argued that GDNF variants are associated with the pathogenesis of OSA via the influence of ventilatory control abnormalities due to the following reasons [8]. First, ventilatory control abnormalities may predispose individuals to OSA by exacerbating ventilatory instability, impairing arousal response to airway obstruction, or promoting an imbalanced activation of the upper airway muscles when compared with the chest wall muscles. Additionally, GDNF is a key factor influencing the development of neural pathways (e.g., development of noradrenergic neurons and differentiation), which are vital for normal respiration. Moreover, GDNF seems to play a pivotal role in sensory afferent neurons in the carotid body, which could be essential in the development of hypoxic responses. Finally, knockout of GDNF gene results in an abnormal central respiratory output and severe mutations in GDNF are associated with the congenital central hypoventilation syndrome. Similarly, the present study revealed that GDNF expression decreased in OSA samples. Notably, the results of Larkin et al. were not successfully replicated in a large Icelandic OSA cohort [30], and the SNPs in the study were not identified in our study, which could be because of the variation in study designs, sample sizes, study populations, or environmental factors in the studies.

In addition, due to the increasingly recognized adverse impacts of OSA on cardiovascular disease (CVD), metabolic syndrome, and neuropsychiatric disorders, Mukherjee et al. [7] suggested that a key subject for consideration in future research should be to identify the genetic links between OSA and adverse health outcomes, that is, to examine if genetic variants are shared between OSA and comorbid disorders. GDNF is one of the most potent neurotrophic factors for catecholaminergic neurons and its involvement in the survival, proliferation, differentiation, and migration of nigrostriatal dopaminergic neurons is well-known [32]. Previous animal studies have demonstrated that GDNF is intimately associated with learning and memory [33, 34] and that its heterozygous mutation (GDNF+/−) in mice causes anomalies in hippocampal synaptic transmission, which suggests the role of GDNF in cognitive performance [35]. Furthermore, GDNF has been reported to be associated with aging [36] and various diseases such as Alzheimer's, Huntington's, and Parkinson's diseases [37], as well as several neuropsychiatric disorders [38]. The role of GDNF in the treatment of Parkinson's disease is currently being investigated through clinical trials [39]. OSA has been strongly associated with CVD, metabolic syndrome, and neuropsychiatric disorders, and some genetic variants could also be critical in determining whether pathways disrupted during the pathogenesis of OSA causally contribute to related consequences [7, 8]. Consequently, it can be presumed that a mutation in the GDNF gene could cause OSA and cognitive dysfunction through different mechanistic effects, which warrants further research.

The present study had several limitations. First, the two microarray datasets used were obtained from public databases, which were of poor quality, and the sample size for targeted sequencing was relatively small due to financial constraints. Consequently, we cannot rule out the fact that lack of statistical power influenced our results; replication studies should be conducted in future using a large sample size. Second, the results of the present study are subject to confounding factors due to the use of different original samples and conditions of the training and validation datasets, and the external validity of our results could be limited. Therefore, more appropriate and robust validations are required in future studies. Third, the number of patients with severe OSA enrolled in the study may not fully represent the general OSA population. Fourth, we did not investigate the interaction between gene and environmental exposure, which underlies the pathogenesis of many complex diseases. Finally, the exact mechanisms of the identified rare nonsynonymous variants and the effect of GDNF gene mutation on the cognitive function of patients with OSA remain indeterminate and require further functional studies.

In conclusion, the present research is a first attempt at studying potential genetic mechanisms influencing OSA through a combination of bioinformatics and targeted capture sequencing analyses. GDNF was identified as a promising candidate gene associated with OSA and its two rare nonsynonymous mutations (c.166G>A–p.Asp56Asn; c.278G>A–p.Arg93Gln), which could be deleterious. Therefore, further functional studies should be conducted to validate their potential role as future diagnostic, prognostic, or therapeutic biomarkers for OSA.

## Figures and Tables

**Table 1:** Top 10 genes by nine ranked methods, respectively, in cytoHubba.

	Rank methods in cytoHubba								
	MCC	Betweenness	Degree	Stress	EcCentricity	BottleNeck	Radiality	Closeness	EPC
TOP 10 genes	SST	SST	SST	SST	PRL	SST	SST	SST	SST
	PRL	EYA1	PRL	EYA1	GDNF	GDNF	PRL	PRL	PRL
	KRTAP20-3	GDNF	SLC2A2	GDNF	SST	PRL	GDNF	GDNF	VIP
	KRTAP19-7	PRL	GDNF	PRL	SLC2A2	RAD21L1	VIP	VIP	BRS3
	KRTAP19-6	SLC2A2	EYA1	SLC2A2	EYA1	SMC1B	SLC2A2	SLC2A2	SLC2A2
	KRTAP3-2	VIP	CHRM2	VIP	GFRAL	EYA1	HOXA11	BRS3	AVPR1B
	HRG	CHRM2	BRS3	TAAR9	NPVF	HOXA11	EYA1	EYA1	GDNF
	SLC2A2	MYF5	VIP	MYF5	UCN3	TRIML1	BRS3	CHRM2	CHRM2
	GDNF	SPAG6	TEX11	SPAG6	HOXA11	ASB5	UCN3	HOXA11	UCN3
	EYA1	TAAR9	PROZ	HOXA11	DCX	SLC2A2	CHRM2	UCN3	HRH4

MCC, Betweenness, Degree, Stress, EcCentricity, BottleNeck, Radiality, Closeness, and EPC are different algorithm names.

**Table 2:** Comparison of baseline data of enrolled subjects from GSE75097.

Variables	Non-OSA (*n* = 6)	OSA (*n* = 6)	*P* value
Gender (male, %)	4 (66.7%)	4 (66.7%)	1.000
Age (years)	51.00 ± 11.35	46.83 ± 9.43	0.505
Hypertension (*n*, %)	2 (33.3%)	2 (33.3%)	1.000
Excessive daytime sleepiness (*n*, %)	4 (66.7%)	4 (66.7%)	1.000
GDNF expression	3.66 (3.04, 3.75)	2.23 (1.55, 2.69)	0.015
Apnea hypopnea index (events/h)	4.17 ± 2.13	47.23 ± 24.33	0.007

**Table 3:** Baseline characteristics of subjects for targeted sequencing.

Variables	Non-OSA (*n* = 50)	Severe OSA (*n* = 50)	*P* value
Gender (male, %)	29 (58.0%)	40 (80.0%)	0.017
Age (years)	44.96 ± 11.44	51.20 ± 10.53	0.006
Body mass index (kg/m^2^)	24.96 (22.96, 26.66)	30.06 (27.45, 31.68)	<0.001
Neck circumference (cm)	38.88 ± 3.62	43.34 ± 3.41	<0.001
Abdomen circumference (cm)	97.78 ± 9.53	110.36 ± 7.83	<0.001
Alcohol history (*n*, %)	14 (28.0%)	7 (14.0%)	0.086
Systolic blood pressure (mmHg)	150.80 ± 21.32	149.44 ± 20.95	0.748
Diastolic blood pressure (mmHg)	94.86 ± 15.64	92.16 ± 15.58	0.389
Apnea hypopnea index (events/h)	0.80 (0.40, 2.13)	59.05 (53.85, 70.60)	<0.001
Apnea index (events/h)	0.00 (0.00, 0.30)	43.25 (32.45, 57.90)	<0.001
Hypopnea index (events/h)	0.75 (0.38, 1.90)	14.75 (9.65, 27.70)	<0.001
Lowest oxyhemoglobin saturation (%)	89.0 (87.0, 91.0)	68.0 (61.0, 74.0)	<0.001
Mean arterial oxygen saturation (%)	94.00 (93.00, 95.25)	91.00 (88.75, 92.00)	<0.001
Sleep efficiency (%)	68.20 (63.20, 74.75)	72.35 (61.10, 78.18)	0.329
Total sleep time (min)	413.25 (379.00, 449.13)	421.50 (387.38, 453.38)	0.608

**Table 4:** Summary of detected rare variants by target sequencing of GDNF.

Position	Ref/Alt allele	Samples	Function	Predicted protein variants	1000G	ExAC03	gnomAD exome	Effect of the mutation				
								SIFT	POLYPhen V2	MutationTaster	CADD raw	DANN
37816009	C/T	1	Nonsynonymous SNV	NM_000514:exon3:c.278G>A:p.R93Q	—	0.00009896	0.00009549	T	P	D	4.723	0.997
37816121	C/T	1	Nonsynonymous SNV	NM_000514:exon3:c.166G>A:p.D56N	—	—	0.000003995	T	P	D	1.555	0.987

GDNF: glial cell-derived neurotrophic factor; 1000G: 1000 Genomes Project; ExAC03: 65000 exome allele frequency data for all; gnomAD: Genome Aggregation Database; SIFT: Sorting Intolerant From Tolerant (T: tolerated); POLYPhen V2: polymorphism phenotyping (P: possibly damaging); MutationTaster: (D: disease_causing); CADD: combined annotation-dependent depletion (cutoff is usually set as 4); DANN: a deep neural network (cutoff is usually set as 0.93).

## Data Availability

The datasets relevant to this study are publicly available from the NCBI GEO database (https://www.ncbi.nlm.nih.gov/geo/query/acc.cgi?acc=GSE135917, https://www.ncbi.nlm.nih.gov/geo/query/acc.cgi?acc=GSE75097).

## Supplementary Materials

Supplementary 1 Figure S1: data preprocessing for GSE135917 (the distribution diagrams of gene expression values before and after normalization). Table S1: functional roles of 4 hub genes (GDNF, SLC2A2, PRL, SST). Table S2: baseline characteristics of enrolled subjects from GSE75097. Table S3: univariate logistic regression analyses of 29 SNPs in GDNF gene with the risk of OSA.

Supplementary 2 Full list of differentially expressed genes; STROBE_checklist_case-control.
