# Supplementary material for: Glial Cell-Derived Neurotrophic Factor Functions as a Potential Candidate Gene in Obstructive Sleep Apnea Based on a Combination of Bioinformatics and Targeted Capture Sequencing Analyses
Source: Biomed Res Int. 2021 Feb 18;2021:6656943. doi: 10.1155/2021/6656943 (PMC7911711; doi:10.1155/2021/6656943)
Supplement: Supplementary 1 — Figure S1: data preprocessing for GSE135917 (the distribution diagrams of gene expression values before and after normalization). Table S1: functional roles of 4 hub genes (GDNF, SLC2A2, PRL, SST). Table S2: baseline characteristics of enrolled subjects from GSE75097. Table S3: univariate logistic regression analyses of 29 SNPs in GDNF gene with the risk of OSA. [file 6656943.f1.doc]

**Supplementary Material 1**

**Supplemental Figure**


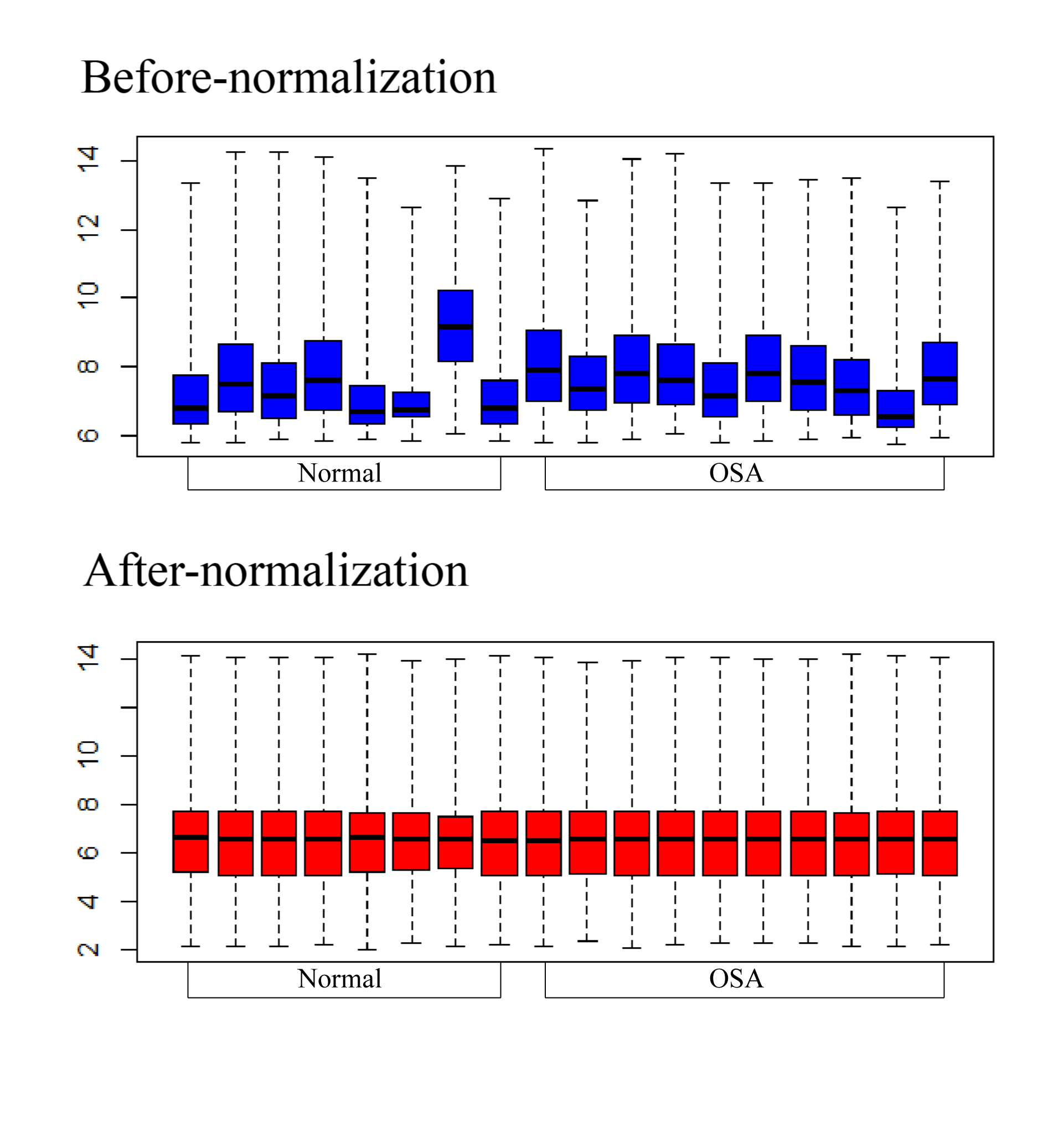


**Figure S1.** Data preprocessing for GSE135917. The distribution diagrams of gene expression values before and after normalization.

**Supplemental Tables**

| **Table S1 Functional roles of 4 hub genes** | | | | | | | | | | |
| --- | --- | --- | --- | --- | --- | --- | --- | --- | --- | --- |
| Gene symbol | Full name | | | | Function | | | | | |
| GDNF | Glial cell derived neurotrophic factor | | | | Neurotrophic factor that enhances survival and morphological differentiation of dopaminergic neurons and increases their high-affinity dopamine uptake | | | | | |
| SLC2A2 | Solute carrier family 2 member 2 | | | | Facilitative glucose transporter | | | | | |
| PRL | Prolactin | | | | Prolactin acts primarily on the mammary gland by promoting lactation | | | | | |
| SST | Somatostatin | | | | Somatostatin inhibits the release of somatotropin | | | | | |
| **Table S2 Baseline characteristics of enrolled subjects from GSE75097** | | | | | | | | | |  |
| Sample | | Group | Apnea hyponea index (events/h) | Gender | | Age | Hypertension | Excessive daytime sleepiness | GDNF expression |  |
| GSM1942596 | | OSA | 26.7 | female | | 52 | Yes | No | 2.1043367 |  |
| GSM1942599 | | OSA | 33.4 | male | | 34 | No | No | 1.3219281 |  |
| GSM1942609 | | OSA | 24.3 | female | | 52 | No | Yes | 2.4854268 |  |
| GSM1942619 | | OSA | 86.5 | male | | 46 | No | Yes | 1.6322682 |  |
| GSM1942623 | | OSA | 48.6 | male | | 59 | Yes | Yes | 2.3504972 |  |
| GSM1942626 | | OSA | 63.9 | male | | 38 | No | Yes | 3.3074285 |  |
| GSM1942595 | | non-OSA | 4.5 | male | | 50 | No | Yes | 3.7441611 |  |
| GSM1942610 | | non-OSA | 2.4 | male | | 36 | No | No | 3.7761040 |  |
| GSM1942616 | | non-OSA | 4.3 | male | | 43 | No | Yes | 2.1699250 |  |
| GSM1942621 | | non-OSA | 8.1 | female | | 59 | Yes | No | 3.6205864 |  |
| GSM1942624 | | non-OSA | 3.3 | male | | 68 | No | Yes | 3.3362834 |  |
| GSM1942633 | | non-OSA | 2.4 | female | | 50 | Yes | Yes | 3.7004397 |  |

| **Table S3 Univariate logistic regression analyses of 29 SNPs in GDNF gene with the risk of OSA** | | | | | | | | | | | | | | | |
| --- | --- | --- | --- | --- | --- | --- | --- | --- | --- | --- | --- | --- | --- | --- | --- |
| SNP ID | Gene Region | Ref Allele | Alt Allele | Freq_Alt (1000g) | Additive | |  | Dominant | |  | Recessive | |  | Allele | |
|  |  |  |  |  | OR(95%CI) | *P* |  | OR(95%CI) | *P* |  | OR(95%CI) | *P* |  | OR(95%CI) | *P* |
| rs17379771 | UTR3 | C | A | 0.235 | 0.941(0.474-1.867) | 0.861 |  | 0.923(0.421-2.022) | 0.841 |  | 1.000(0.127-7.872) | 0.999 |  | 0.951(0.511-1.769) | 0.874 |
| rs11111 | UTR3 | T | C | 0.248 | 1.121(0.577-2.177) | 0.735 |  | 1.275(0.579-2.807) | 0.547 |  | 0.653(0.104-4.085) | 0.648 |  | 1.113(0.586-2.111) | 0.744 |
| rs3749692 | UTR3 | A | G | 0.540 | 0.956(0.532-1.718) | 0.881 |  | 0.583(0.177-1.925) | 0.376 |  | 1.185(0.528-2.660) | 0.680 |  | 0.958(0.541-1.699) | 0.884 |
| rs2973050 | intronic | A | G | 0.604 | 0.910(0.499-1.661) | 0.759 |  | 1.000(0.332-3.013) | 0.999 |  | 0.820(0.342-1.966) | 0.656 |  | 0.922(0.527-1.612) | 0.776 |
| rs13361833 | intronic | C | T | 0.160 | 0.750(0.384-1.464) | 0.399 |  | 0.850(0.386-1.874) | 0.687 |  | 0.235(0.025-2.178) | 0.202 |  | 0.763(0.399-1.457) | 0.412 |
| rs2910797 | intronic | C | T | 0.329 | 0.892(0.417-1.907) | 0.768 |  | 1.206(0.460-3.165) | 0.703 |  | 0.220(0.023-2.079) | 0.186 |  | 0.889(0.411-1.922) | 0.765 |
| rs5867365 | intronic | - | T | 0.329 | 0.750(0.384-1.464) | 0.399 |  | 0.852(0.388-1.868) | 0.689 |  | 0.235(0.025-2.178) | 0.202 |  | 0.773(0.412-1.452) | 0.424 |
| rs1549250 | intronic | C | A | 0.476 | 1.309(0.686-2.500) | 0.414 |  | 1.176(0.534-2.593) | 0.687 |  | 2.667(0.492-4.445) | 0.255 |  | 1.261(0.693-2.294) | 0.447 |
| rs2910706 | intronic | C | A | 0.163 | 0.833(0.420-1.653) | 0.602 |  | 0.922(0.419-2.028) | 0.840 |  | 0.320(0.032-3.184) | 0.331 |  | 0.850(0.446-1.621) | 0.662 |
| rs2973046 | intronic | T | G | 0.328 | 0.833(0.420-1.653) | 0.601 |  | 0.923(0.421-2.022) | 0.841 |  | 0.320(0.032-3.184) | 0.331 |  | 0.857(0.457-1.608) | 0.631 |
| rs2973045 | intronic | G | A | 0.326 | 0.794(0.407-1.549) | 0.499 |  | 0.923(0.421-2.022) | 0.841 |  | 0.235(0.025-2.178) | 0.202 |  | 0.816(0.437-1.526) | 0.524 |
| rs2910705 | intronic | T | C | 0.164 | 0.750(0.384-1.464) | 0.399 |  | 0.850(0.386-1.874) | 0.687 |  | 0.235(0.025-2.178) | 0.202 |  | 0.763(0.399-1.457) | 0.412 |
| rs1104682 | intronic | G | A | 0.163 | 0.750(0.384-1.464) | 0.399 |  | 0.850(0.386-1.874) | 0.687 |  | 0.235(0.025-2.178) | 0.202 |  | 0.763(0.399-1.457) | 0.412 |
| rs2973043 | intronic | A | G | 0.826 | 0.863(0.466-1.597) | 0.639 |  | 0.468(0.110-1.987) | 0.303 |  | 1.000(0.443-2.258) | 0.999 |  | 0.872(0.482-1.577) | 0.651 |
| rs884344 | intronic | A | C | 0.333 | 0.750(0.384-1.464) | 0.399 |  | 0.852(0.388-1.868) | 0.689 |  | 0.235(0.025-2.178) | 0.202 |  | 0.773(0.412-1.452) | 0.424 |
| rs2973042 | intronic | T | G | 0.384 | 0.756(0.414-1.381) | 0.363 |  | 0.711(0.316-1.601) | 0.411 |  | 0.683(0.201-2.315) | 0.539 |  | 0.773(0.435-1.374) | 0.379 |
| rs2910702 | intronic | C | T | 0.737 | 0.910(0.498-1.662) | 0.759 |  | 0.638(0.168-2.413) | 0.508 |  | 1.000(0.443-2.258) | 0.999 |  | 0.913(0.507-1.647) | 0.764 |
| rs2910701 | intronic | C | T | 0.164 | 0.750(0.384-1.464) | 0.399 |  | 0.850(0.386-1.874) | 0.687 |  | 0.235(0.025-2.178) | 0.202 |  | 0.763(0.399-1.457) | 0.412 |
| rs2973041 | intronic | T | C | 0.263 | 0.765(0.424-1.379) | 0.373 |  | 0.719(0.324-1.597) | 0.418 |  | 0.683(0.201-2.315) | 0.539 |  | 0.768(0.429-1.375) | 0.374 |
| rs1862574 | intronic | A | C | 0.737 | 0.910(0.498-1.662) | 0.759 |  | 0.638(0.168-2.413) | 0.508 |  | 1.000(0.443-2.258) | 0.999 |  | 0.913(0.507-1.647) | 0.764 |
| rs12518844 | intronic | G | A | 0.312 | 1.275(0.642-2.531) | 0.487 |  | 1.086(0.489-2.411) | 0.839 |  | 4.261(0.459-9.546) | 0.202 |  | 1.202(0.663-2.179) | 0.544 |
| rs3096140 | intronic | G | A | 0.700 | 0.833(0.459-1.509) | 0.546 |  | 0.534(0.146-1.954) | 0.343 |  | 0.922(0.417-2.035) | 0.839 |  | 0.837(0.466-1.503) | 0.551 |
| rs10941370 | intronic | T | C | 0.482 | 1.114(0.584-2.124) | 0.742 |  | 1.185(0.528-2.660) | 0.680 |  | 1.000(0.225-4.439) | 0.999 |  | 1.092(0.611-1.953) | 0.767 |
| rs2973038 | intronic | C | G | 0.720 | 0.957(0.535-1.712) | 0.882 |  | 1.000(0.288-3.470) | 0.999 |  | 0.922(0.417-2.035) | 0.839 |  | 0.957(0.534-1.714) | 0.882 |
| rs57148382  (STR) | intronic | TT | - | 0.133 | 0.944(0.487-1.833) | 0.866 |  | 1.000(0.443-2.258) | 0.999 |  | 0.653(0.104-4.085) | 0.648 |  | 0.947(0.497-1.805) | 0.869 |
| rs2973036 | intronic | G | C | 0.380 | 0.795(0.408-1.548) | 0.499 |  | 0.786(0.358-1.726) | 0.548 |  | 0.653(0.104-4.085) | 0.648 |  | 0.812(0.431-1.530) | 0.519 |
| rs2910700 | intronic | C | T | 0.195 | 1.000(0.494-2.022) | 0.999 |  | 1.000(0.443-2.258) | 0.999 |  | 1.000(0.127-7.872) | 0.999 |  | 1.000(0.512-1.953) | 0.999 |
| rs2973035 | intronic | G | A | 0.157 | 0.885(0.446-1.756) | 0.727 |  | 0.848(0.383-1.879) | 0.685 |  | 1.000(0.127-7.872) | 0.999 |  | 0.893(0.462-1.727) | 0.737 |
| rs2973033 | UTR5 | T | C | 0.405 | 0.734(0.389-1.383) | 0.339 |  | 0.613(0.277-1.358) | 0.228 |  | 1.000(0.225-4.439) | 0.999 |  | 0.759(0.418-1.377) | 0.364 |
| GDNF: glial cell derived neurotrophic factor; SNP: single nucleotide polymorphism; Ref: Reference; Alt: Alternate; UTR: untranslated region; STR: short tandem repeat; Freq_Alt (1000g): the frequency of alternative allele in 1000 Genomes Project; OR: odds ratio; CI: confidence interval. | | | | | | | | | | | | | | | |
